# Thickness-Dependent Optical Properties and Exciton Recombination Dynamics in the GaSe_0.5_Te_0.5_ Alloy

**DOI:** 10.1021/acsomega.6c03781

**Published:** 2026-07-15

**Authors:** Maykol Oliveira, Fabrício M. de Vasconcelos, Fábio F. Leite, Nestor Perea-López, Da Zhou, Mauricio Terrones, Francisco E. P. Santos, Antonio G. Souza Filho, Bartolomeu C. Viana, Rafael S. Alencar

**Affiliations:** † Departamento de Física, 67823 Universidade Federal Do Piauí, Teresina, Piauí 64049-550, Brazil; ‡ 119529 Instituto Federal Do Piauí - Campus Piripiri, Piripiri, Piauí 64260-000, Brazil; § Departamento de Ciência Exatas E Tecnológicas, 74364 Universidade Federal Do Amapá, Macapá, Amapá 68903-419, Brazil; ∥ Department of Physics, 311285 The Pennsylvania State University, University Park, Pennsylvania 16802, United States; ⊥ Center for 2-Dimensional and Layered Materials, The Pennsylvania State University, University Park, Pennsylvania 16802, United States; # Department of Chemistry, The Pennsylvania State University, University Park, Pennsylvania 16802, United States; ∇ Department of Materials Science and Engineering, The Pennsylvania State University, University Park, Pennsylvania 16802, United States; ○ Pós-Graduação em Ciência e Engenharia dos Materiais, Universidade Federal Do Piauí, Teresina, Piauí 64049-550, Brazil; ◆ Departamento de Física, 28121 Universidade Federal Do Ceará, Fortaleza, Ceará 60455-900, Brazil

## Abstract

In this study, we investigate the thickness-dependent optical properties of GaSe_0.5_Te_0.5_ alloy by combining Raman spectroscopy, photoluminescence (PL) measurements, and density functional theory (DFT) calculations. Raman measurements reveal thickness-dependent variations in phonon frequencies and intensities below ∼20 nm, enabling thickness identification through frequency differences between phonon modes. In addition, PL measurements show a pronounced quenching of the PL intensity with decreasing thickness, attributed to enhanced nonradiative recombination associated with surface states and thickness-induced modifications of the electronic structure, accompanied by a blueshift of the PL energy. In agreement with these observations, first-principles calculations demonstrate a thickness-driven evolution of the band structure, with a transition from an indirect character in few-layer systems toward a more direct gap in thicker structures, governed by the interplay between quantum confinement and interlayer coupling. Complementary excitation-power-dependent PL measurements further show distinct recombination dynamics in bulk samples, with sublinear behavior for trapped excitons and superlinear dependence for bound and free excitons, consistent with a state-filling mechanism in localized states. These findings establish a consistent framework linking thickness, electronic structure, and recombination processes in GaSe_0.5_Te_0.5_ alloy, and provide key insights for the design and optimization of optoelectronic devices based on PTMC alloys.

## Graphical abstract

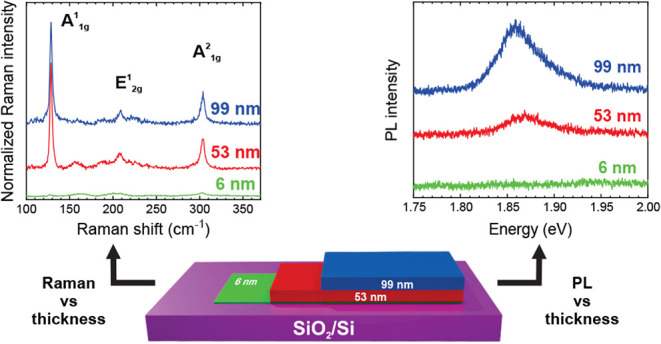

## Introduction

Two-dimensional (2D) materials have attracted significant attention due to their unique electronic and optical properties arising from reduced dimensionality and weak interlayer interactions.^1^ Since the isolation of graphene,^2^ a wide variety of layered systems has been explored, including black phosphorus, hexagonal boron nitride, transition-metal dichalcogenides, topological insulators, and post-transition-metal chalcogenides (PTMCs).^3–14^

PTMCs form an important class of layered materials, generally structured in layers where two bonded metal atoms are enveloped by two chalcogen atoms.^13–17^ Among them, gallium (Ga) monochalcogenides stand out as van der Waals semiconductors with an adjustable band gap, efficient light absorption, and high charge carrier mobility, making them promising for photonic and optoelectronic applications.^11^ Bulk GaSe exhibits an indirect band gap of approximately 2.1 eV^18^ and is widely used in nonlinear optical systems as well as in photodetectors and sensors.^19–22^ In contrast, bulk GaTe has a direct band gap of 1.65 eV with higher light absorption, which makes it particularly attractive for optoelectronic applications.^23,24^

These distinct electronic characteristics can be systematically controlled through alloy engineering. Specifically, GaSe_1–*x*_Te_*x*_ alloys are known to be stable over a wide compositional range, with a band gap strongly dependent on *x*.^25,26^ However, achieving precise control over structural properties, composition, and defect states remains a significant challenge even in alloyed systems. In addition to compositional tuning, thickness plays a key role in defining the optical and vibrational properties of these systems.^27^ Reduced dimensionality enhances quantum confinement and weakens dielectric screening, leading to stronger many-body interactions and the formation of tightly bound excitons.^28–30^ These excitonic states can exhibit complex relaxation pathways and strong coupling to lattice vibrations, playing a fundamental role in excitonic recombination and optical processes in layered semiconductors.^31–34^ Despite recent studies on bulk GaSe_0.5_Te_0.5_ addressing phonon dynamics and photoluminescence under external perturbations,^13,14^ a systematic understanding of how thickness governs vibrational properties, optical emission, and exciton recombination mechanisms in this alloy remains lacking.

In this work, we report thickness-dependent optical properties of the GaSe_0.5_Te_0.5_ alloy by combining Raman spectroscopy, PL measurements, and DFT calculations. We examine how dimensionality influences the vibrational response and optical emission, while exciton recombination mechanisms are further assessed through complementary excitation-power-dependent measurements. This approach enables us to elucidate the interplay between quantum confinement, interlayer coupling, and localized states, establishing a unified picture of the thickness-dependent optical behavior of PTMC alloys.

## Results and Discussion

Raman spectroscopy is a fast and useful technique for identifying the number of layers in 2D materials due to the strong thickness dependence of their Raman signatures.^35–41^ The Raman spectrum of bulk GaSe_0.5_Te_0.5_ is characterized by three main peaks with phonon frequencies (irreducible representations) at 128.3 cm^–1^ $\left(A_{1g}^{1}\right)$ , 208.0 cm^–1^ $\left(E_{2g}^{1}\right)$ , and 303.2 cm^–1^ $\left(A_{1g}^{2}\right)$ .^14^ Unlike typical PTMCs^37,39,42^ and TMDCs,^35^ where the *A*_1*g*_ and *E*_2*g*_ modes display purely out-of-plane and in-plane vibrations, respectively, the *A*_1*g*_ and *E*_2*g*_ modes in the GaSe_0.5_Te_0.5_ alloy present small mixed components along both directions.^14^

Figure 1a shows the thickness dependence of the Raman modes $A_{1g}^{1},\;$ $E_{2g}^{1}$ , and $A_{1g}^{2}$ . For a more accurate analysis, each mode was fitted using a single Lorentzian component, and the corresponding thickness-dependent frequency and intensity are presented in Figure 1b and c, respectively. Overall, the vibrational modes exhibit a significant decrease in intensity as the thickness is reduced from 20 nm. Furthermore, below 20 nm, the frequencies of the $A_{1g}^{1}$ and $E_{2g}^{1}$ modes increase, while that of the $A_{1g}^{1}.$ mode decreases. These frequency shifts are more clearly evidenced by considering the differences between the mode frequencies, as shown in Figure 1b, which acts as a reliable thickness indicator, similar to what has been reported for TMDs^43,44^ and TMMs.^45–47^

**Figure 1:**
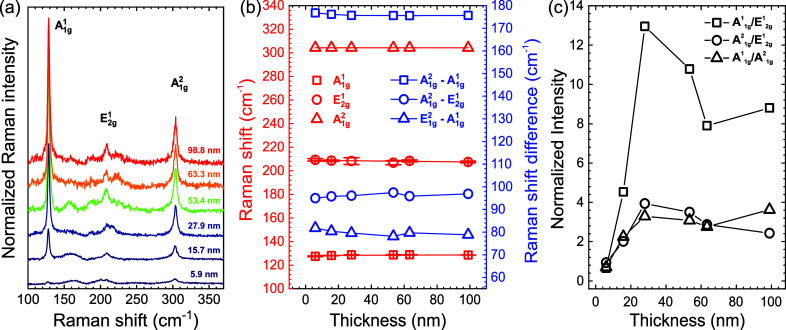
(a) Raman spectra of GaSe_0.5_Te_0.5_ alloy for different thicknesses. (b) Thickness dependence of the Raman mode frequencies $A_{1g}^{1}$ , $E_{2g}^{1}$ , and $A_{1g}^{2}$ (left red axis) and their frequency differences $A_{1g}^{2}-A_{1g}^{1}$ , $A_{1g}^{2}-E_{2g}^{1}$ , and $E_{2g}^{1}-A_{1g}^{1}$ (right blue axis). Open red squares, circles, and triangles represent the Raman modes, while open blue squares, circles, and triangles represent their differences, respectively. Red and blue lines are guides to the eye. (c) Intensity ratios $A_{1g}^{1}/E_{2g}^{1}$ , $A_{1g}^{2}/E_{2g}^{1}$ , and $A_{1g}^{1}/A_{1g}^{2}$ , represented by open black squares, circles, and triangles, respectively, as a function of thickness. Black lines are guides to the eye.

At ambient conditions, the bulk GaSe_0.5_Te_0.5_ alloy exhibits an asymmetric PL band that can be well fitted using two Voigt components centered at 1.87 eV (blue) and 1.89 eV (purple), as shown in Figure 2a. These features arise, respectively, from the direct free-exciton (FE) transition at the Γ point and an indirect transition (IT) from the valence band maximum at Γ to the conduction band minimum at the high-symmetry point Q.^13^ At 90 K, a broad band appears on the low-energy side of the direct transition (Figure 2b), which is attributed to recombination involving localized excitonic states, commonly associated with bound (BE) and trapped (TE) excitons in PTMCs.^13,48–50^ Bound excitons are associated with shallow localized states and therefore remain energetically close to the free-exciton transition, whereas trapped excitons originate from deeper localization potentials, resulting in emission at lower energies. In PTMC alloys, both types of localized states may arise from defects, impurities, and local compositional fluctuations.

**Figure 2:**
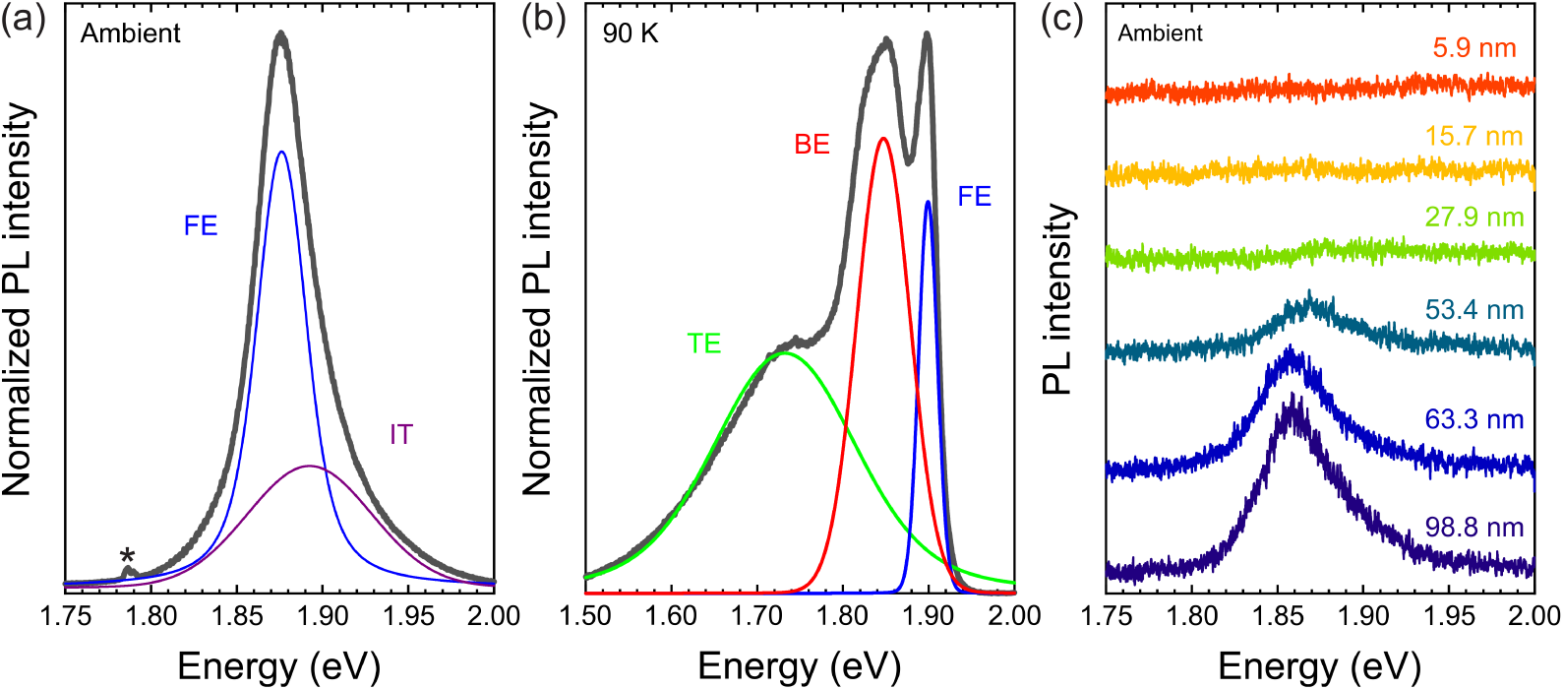
(a) PL spectrum of bulk GaSe_0.5_Te_0.5_ at ambient conditions, showing contributions from the direct (free exciton) transition (blue trace) and the indirect transition (purple trace). (b) PL spectrum at 90 K, where additional features associated with trapped (light green trace) and bound (red trace) exciton emissions are observed. (c) Thickness-dependent PL spectra of GaSe_0.5_Te_0.5_.

Figure 2c shows the thickness dependence of the PL signals. Similar to GaSe,^48^ GaTe,^51^ and InSe, the PL intensity strongly decreases as the thickness is reduced, accompanied by a blueshift of the PL peak energy. This pronounced PL quenching can be attributed to a combination of two effects: (i) a reduction of the internal quantum yield due to enhanced nonradiative recombination associated with surface states^48^ and (ii) intrinsic electronic-structure modifications induced by quantum confinement, leading to an evolution toward a more indirect band gap with decreasing thickness.^51^ Although the relative contribution of each mechanism cannot be quantitatively disentangled within the present experimental framework, surface-assisted nonradiative processes are expected to play a dominant role in the ultrathin regime.

To clarify the role of the thickness-dependent electronic structure in the optical response, first-principles calculations were performed. In Figure 3a, we present the top view of the optimized geometric structure of GaSe_0.5_Te_0.5_, with the structure of a rhombus representing the unit cell of the system, in black. The mono-, tri-, six-, and ten-layer GaSe_0.5_Te_0.5_ configurations are illustrated in Figure 3b. All systems were stacked following the most stable AB stacking configuration reported in ref 52.

**Figure 3:**
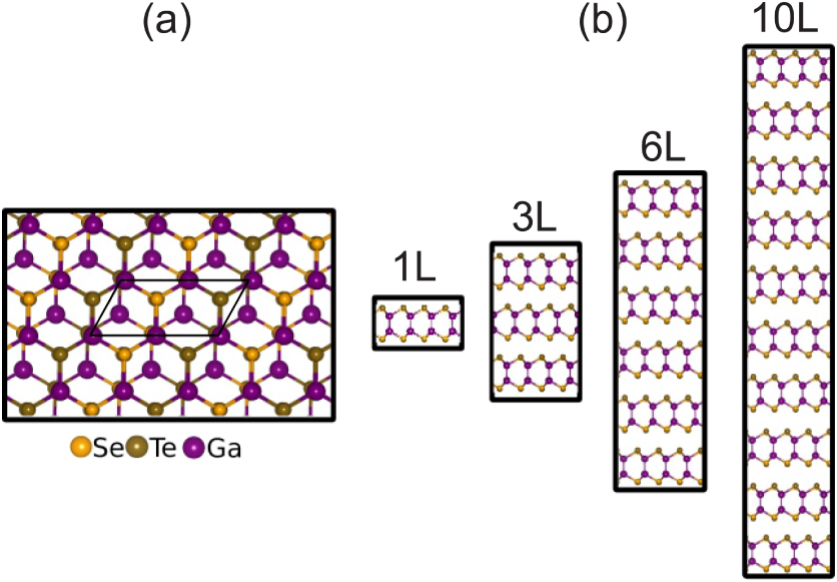
(a) Top view of the atomic structure of GaSe_0.5_Te_0.5_. (b) Side views of mono-, tri-, six-, and ten-layer GaSe_0.5_Te_0.5_ in AB stacking configuration. Selenium, tellurium, and gallium atoms are shown in orange, brown, and purple, respectively.

In Figure 4, we show the electronic band structures of the systems, calculated along the high-symmetry path Γ - M* - *M* - Γ - *A* - Γ of the Brillouin zone (BZ), the valence and conduction band 3D dispersions over the entire BZ, the indirect and direct electronic transitions, and the difference between them.

**Figure 4:**
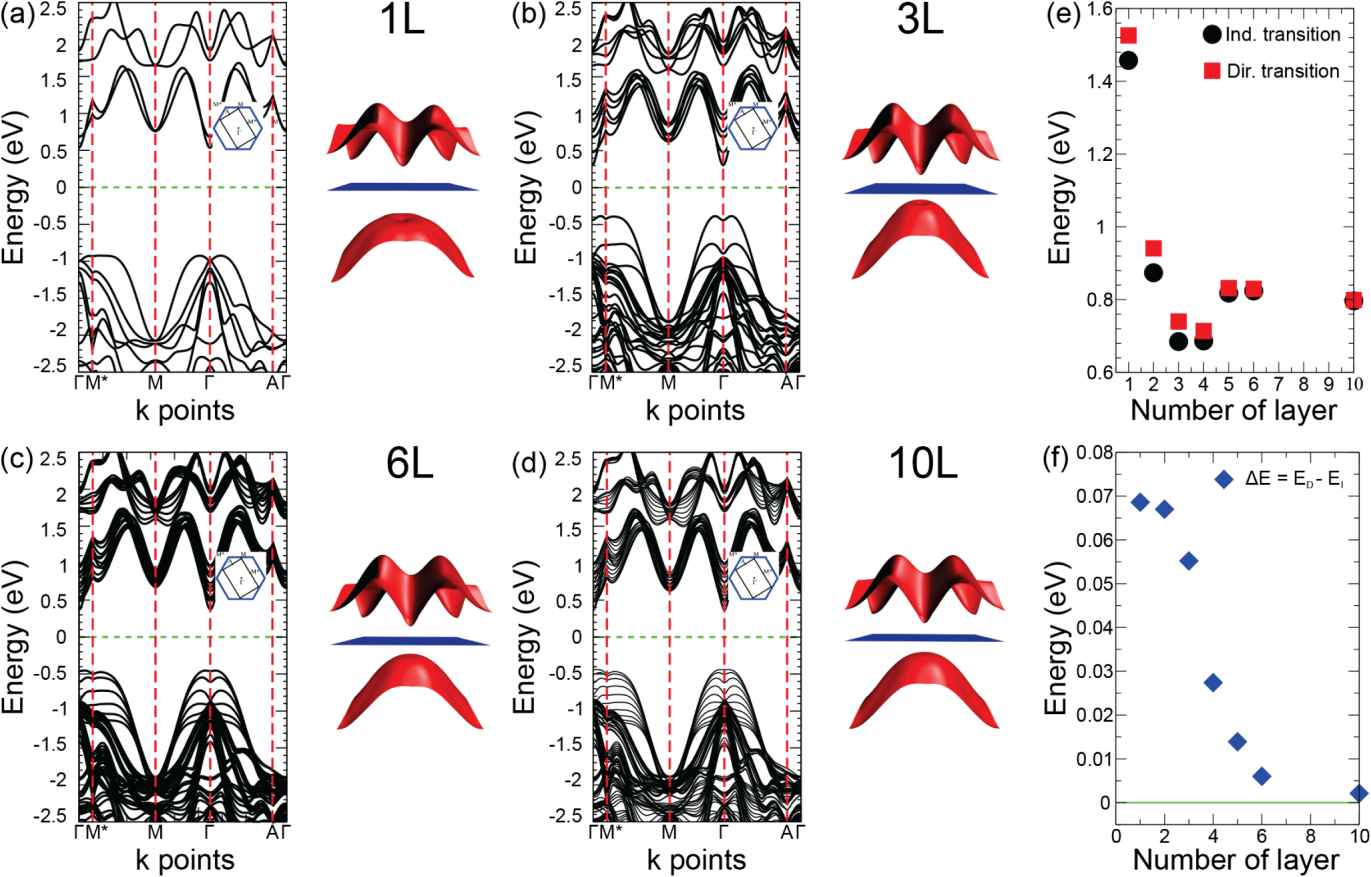
Electronic band structures of GaSe_0.5_Te_0.5_ calculated along selected high-symmetry directions in the BZ, with the Fermi level set to *E* = 0, for (a) monolayer, (b) trilayer, (c) six-layer, and (d) ten-layer systems. The corresponding valence and conduction bands are also shown as red surfaces over the entire BZ. (e) Thickness dependence of the indirect (black circles) and direct (red squares) transitions. (f) Difference between the indirect and direct transitions (blue diamonds) as a function of thickness. The horizontal light green line indicates the zero-energy reference.

While the low-energy region of the conduction band remains nearly parabolic for all thicknesses, with the conduction band minimum (CBM) at the Γ point, the valence band exhibits an inverted Mexican-hat-like dispersion in the monolayer and few-layer limit,^53^ with the valence band maximum (VBM) located at saddle points away from the Γ point, as demonstrated in Figure 4a–b. This feature enhances the indirect character of the band gap in ultrathin layers. As the number of layers increases, the Mexican-hat character of the VBM progressively disappears, and the valence band evolves toward a simple parabolic shape, indicating a transition toward a more direct-gap configuration, as represented in Figure 4c–d.

This evolution reflects the interplay between quantum confinement and interlayer coupling effects. In the monolayer limit, confinement dominates and lifts the band degeneracy, whereas with increasing thickness, interlayer hybridization progressively restores the valence states at the Γ point. To further quantify this indirect-to-direct crossover, Figure 4e shows the evolution of the direct and indirect transitions as a function of thickness. Figure 4f displays the energy difference between the direct and indirect transitions (Δ*E*), which decreases from approximately 0.068 eV in the monolayer to about 0.002 eV in the ten-layer structure. This systematic reduction of Δ*E* highlights the increasing role of interlayer interactions in thicker systems and provides a quantitative indicator of the thickness-driven evolution of the band gap character.

To gain further insight into the recombination mechanisms responsible for the different emission bands observed in the PL spectra, we investigate the excitation power dependence of the photoluminescence of bulk GaSe_0.5_Te_0.5_ at 90 K. This approach is widely used to identify dominant radiative recombination processes and distinguish between competing emission channels.^48,49,54^ Figure 5a shows the GaSe_0.5_Te_0.5_ PL spectra at different excitation powers. At low power, the emission is dominated by the TE band. As the excitation power increases, two higher-energy features progressively emerge, corresponding to the BE transition near 1.83 eV and the FE emission at ∼1.89 eV.

**Figure 5:**
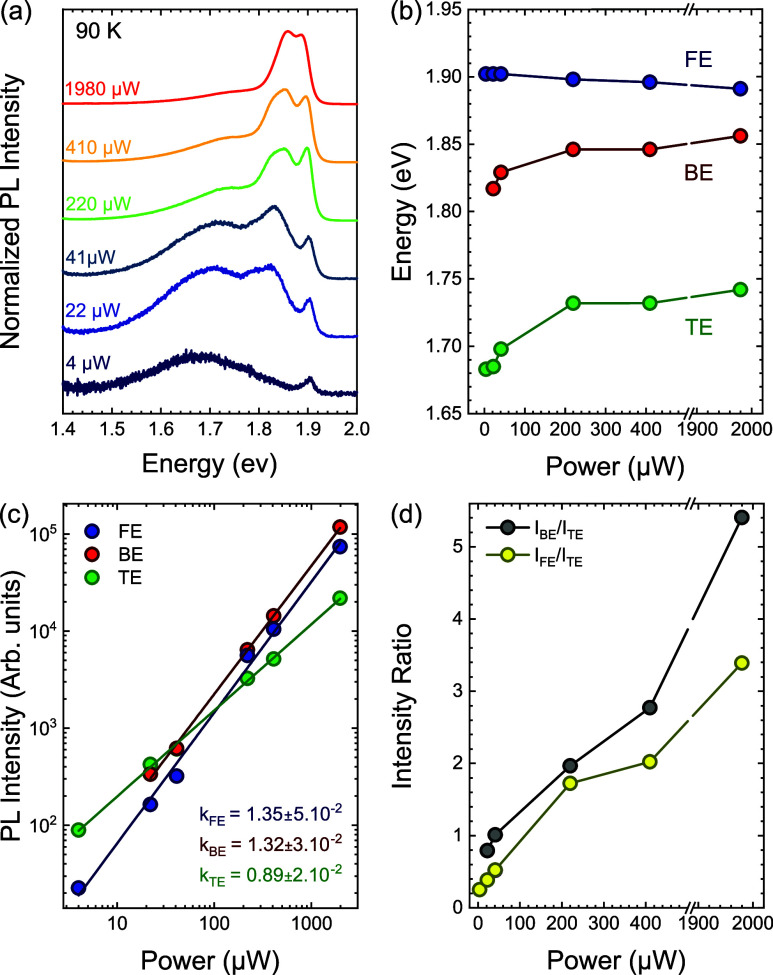
(a) Excitation power dependence of the PL spectra of bulk GaSe_0.5_Te_0.5_ at 90 K. (b) Excitation power dependence of the emission energies of free (blue circles), bound (red circles), and trapped (light green circles) excitons. Solid lines are guides to the eye. (c) Power dependence of the integrated PL intensities of the FE, BE, and TE emissions. The solid lines represent least-squares fits to the data using *I* ∝ *P^k^*, with the corresponding *k* values indicated. (d) Intensity ratios *I*_BE_/*I*_TE_ (dark gray circles) and *I*_FE_/*I*_TE_ (yellow circles) as a function of excitation power. Solid lines are guides to the eye.

Figure 5b shows the power-dependent energy shifts of the TE, BE, and FE extracted from Figure 5a. As the excitation power increases, the FE transition redshifts. Such behavior is consistent with the temperature-dependent redshift previously reported for FE excitation in bulk GaSe_0.5_Te_0.5_ and is attributed to the band gap renormalization associated with electron–phonon coupling and lattice expansion.^13^ In contrast, the BE and TE bands display a clear blueshift with increasing excitation power. Such behavior is characteristic of a state-filling mechanism in localized states, where deeper localized states progressively saturate and recombination shifts toward shallower states.^48,49,54^ In this context, the nonideal stoichiometry of GaSe_0.5_Te_0.5_, as previously reported,^14^ introduces compositional disorder and defect-induced potential fluctuations, providing a natural origin for the distribution of localized states associated with the TE and BE emissions.

The excitation-power dependence of the integrated PL intensities supports this interpretation. The integrated PL intensity, *I*, follows a power-law dependence on the excitation power, *P*, expressed as *I* ∝ *P* ^*k*^, where *k* reflects the dominant recombination mechanism.^40,55–57^ As shown in Figure 5c, the TE emission exhibits a sublinear behavior (*k*_TE_ = 0.89), whereas BE and FE display superlinear behavior (*k*_BE_ ≈ 1.32 and *k*_FE_ ≈ 1.35). While defect-related emission is typically sublinear and free-exciton recombination is expected to scale approximately linearly with excitation power, deviations from this simplified picture arise when multiple recombination channels compete for the same photogenerated carriers.^48,49,54^ At low excitation power, carriers are efficiently captured by deep localized states, leading to dominant TE emission. As the excitation power increases, these states progressively saturate, reducing carrier trapping and enhancing recombination through the BE and FE channels. This redistribution of carriers explains the sublinear response of TE together with the superlinear increase observed for BE and FE. Consistently, Figure 5d shows that both *I*_BE_/*I*_TE_ and *I*_FE_/*I*_TE_ increase with excitation power, confirming the progressive transfer of recombination toward higher-energy channels.

## Conclusion

In conclusion, our results showed that thickness plays a key role in governing the optical properties of GaSe_0.5_Te_0.5_. Raman measurements revealed that variations in phonon modes provide a reliable parameter for thickness identification below ∼20 nm. In addition, PL measurements showed a pronounced quenching of the PL intensity with decreasing thickness, accompanied by a blueshift of the PL energy. These observations reflected the combined influence of enhanced nonradiative recombination and thickness-induced modifications of the electronic structure. Consistently, first-principles calculations indicated a thickness-driven evolution of the band structure from an indirect character in few-layer systems toward a more direct gap in thicker structures. Furthermore, excitation-power-dependent PL measurements performed on bulk GaSe_0.5_Te_0.5_ at 90 K revealed that recombination dynamics were controlled by competing recombination channels, with sublinear behavior for trapped excitons and superlinear dependence for bound and free excitons, consistent with a state-filling mechanism. Overall, these results provide a basis for controlling the optical response of PTMC alloy systems, with direct implications for the design of optoelectronic devices.

## Methodology

### Sample and Experimental Setup

Few-layer GaSe_0.5_Te_0.5_ flakes were mechanically exfoliated from bulk crystals by using adhesive tape and transferred onto cleaned SiO_2_/Si substrates. Subsequently, atomic force microscopy (AFM) topographic images were acquired to accurately determine the thicknesses of the exfoliated regions (Figure 6). Thickness values were extracted from height profiles along six regions, indicated by dashed white lines, resulting in thicknesses of 99, 63, 53, 28, 16, and 6 nm.

**Figure 6:**
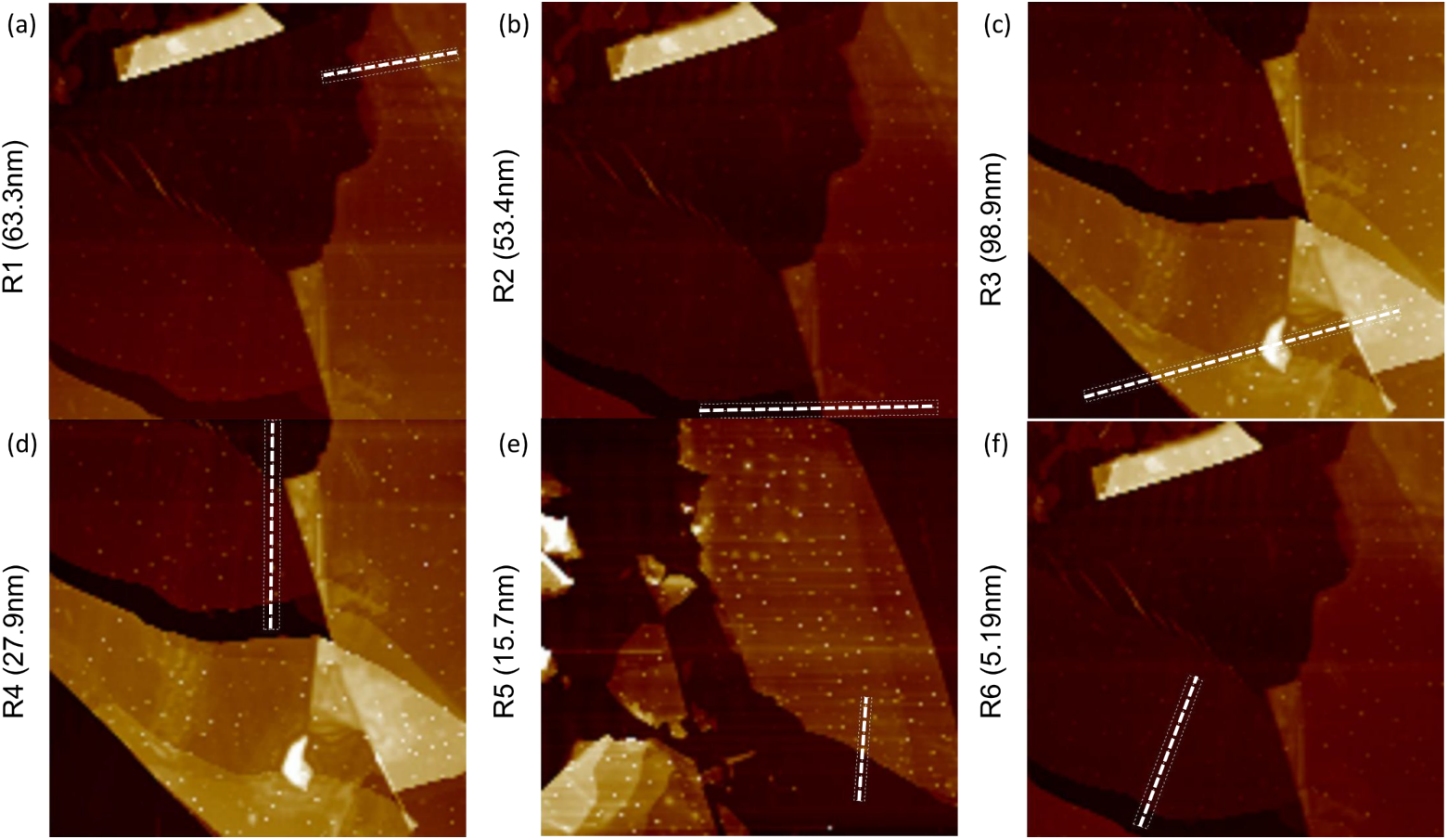
(a–f) AFM topographic images of exfoliated GaSe_0.5_Te_0.5_ flakes. The thickness of each region was determined from the height profile extracted along the white dashed line indicated in each image.

Thickness-dependent Raman and PL spectroscopy experiments were conducted by using a LabRam Evolution system equipped with a confocal microscope, a 100x objective, and a 532 nm excitation laser. For power-dependent PL measurements, a Renishaw InVia spectrometer with 488 nm excitation was employed across six different power levels using a cryogenic stage to maintain the sample temperature at 90 K.

### Density Functional Theory Calculations

To support the interpretation of the thickness-dependent PL response, we performed DFT calculations to investigate the evolution of the electronic band structure as a function of the layer number in the GaSe_0.5_Te_0.5_ alloy. In particular, we focused on the modification of the frontier energy levels with increasing thickness.

Electronic structure calculations were carried out within the DFT framework as implemented in the SIESTA code.^58^ The exchange–correlation energy was described using the Perdew–Burke–Ernzerhof (PBE) functional within the generalized gradient approximation (GGA).^59^ A double-ζ plus polarization (DZP) basis set was employed to expand the valence wave functions, and ionic cores were described using Optimized Norm-Conserving Vanderbilt (ONCV) pseudopotentials.^60^ van der Waals interactions were accounted for using the DFT-D2 method of Grimme.^61,62^

Brillouin zone integrations were performed using a Monkhorst–Pack grid of 16 × 32 × 1 k-points.^63^ A vacuum spacing of 20 Å was introduced along the out-of-plane direction to avoid spurious interactions between periodic images. The real-space grid was defined with a mesh cutoff of 400 Ry. Structural relaxations were carried out using the conjugate gradient method until the maximum force on each atom was below 10^–2^ eV/Å and the maximum stress tensor component was below 0.1 GPa. The convergence criterion for the density matrix was set to less than 10^–4^.
